# Smartphone and Mobile Health Apps for Tinnitus: Systematic Identification, Analysis, and Assessment

**DOI:** 10.2196/21767

**Published:** 2020-08-18

**Authors:** Muntazir Mehdi, Michael Stach, Constanze Riha, Patrick Neff, Albi Dode, Rüdiger Pryss, Winfried Schlee, Manfred Reichert, Franz J Hauck

**Affiliations:** 1 Institute of Distributed Systems Ulm University Ulm Germany; 2 Institute of Databases and Information Systems Ulm University Ulm Germany; 3 Department of Psychology University of Zurich Zurich Switzerland; 4 Clinic and Policlinic for Psychiatry and Psychotherapy Regensburg Germany; 5 URPP Dynamics of Healthy Aging University of Zurich Zurich Switzerland; 6 Institute of Clinical Epidemiology and Biometry University of Würzburg Würzburg Germany

**Keywords:** Health care, Mobile Health, Smartphone Apps, Mobile Apps, Tinnitus, App Quality Assessment and Evaluation, MARS

## Abstract

**Background:**

Modern smartphones contain sophisticated high-end hardware features, offering high computational capabilities at extremely manageable costs and have undoubtedly become an integral part in users' daily life. Additionally, smartphones offer a well-established ecosystem that is easily discoverable and accessible via the marketplaces of differing mobile platforms, thus encouraging the development of many smartphone apps. Such apps are not exclusively used for entertainment purposes but are also commonplace in health care and medical use. A variety of those health and medical apps exist within the context of tinnitus, a phantom sound perception in the absence of any physical external source.

**Objective:**

In this paper, we shed light on existing smartphone apps addressing tinnitus by providing an up-to-date overview.

**Methods:**

Based on PRISMA guidelines, we systematically searched and identified existing smartphone apps on the most prominent app markets, namely Google Play Store and Apple App Store. In addition, we applied the Mobile App Rating Scale (MARS) to evaluate and assess the apps in terms of their general quality and in-depth user experience.

**Results:**

Our systematic search and screening of smartphone apps yielded a total of 34 apps (34 Android apps, 26 iOS apps). The mean MARS scores (out of 5) ranged between 2.65-4.60. The Tinnitus Peace smartphone app had the lowest score (mean 2.65, SD 0.20), and Sanvello—Stress and Anxiety Help had the highest MARS score (mean 4.60, SD 0.10). The interrater agreement was substantial (Fleiss κ=0.74), the internal consistency was excellent (Cronbach α=.95), and the interrater reliability was found to be both high and excellent—Guttman λ6=0.94 and intraclass correlation, ICC(2,k) 0.94 (95% CI 0.91-0.97), respectively.

**Conclusions:**

This work demonstrated that there exists a plethora of smartphone apps for tinnitus. All of the apps received MARS scores higher than 2, suggesting that they all have some technical functional value. However, nearly all identified apps were lacking in terms of scientific evidence, suggesting the need for stringent clinical validation of smartphone apps in future. To the best of our knowledge, this work is the first to systematically identify and evaluate smartphone apps within the context of tinnitus.

## Introduction

Tinnitus is a condition mainly associated with the perception of a continuous ringing noise in the ears in the absence of any external sound source. The direct causative factors of the perception of subjective tinnitus are manifold and are not fully understood. However, tinnitus is often associated with underlying damage in the inner ear, such as the loss of cochlear hair cells. Worldwide, roughly 15% of the population suffers from tinnitus; among them 2% experience a substantial decrease in quality of life due to the phantom percept [1]. At present, tinnitus is considered to be a condition that involves changes at different levels of the auditory pathway, the auditory cortex, and nonauditory areas such as the limbic system. These changes may additionally be influenced by psychosocial stress (eg, negative thoughts, stress at home, increased workload, etc), which affects not only the emotional status of the patient but also the auditory system [2,3]. Consequently, people with tinnitus often report that their perception of it varies, including its loudness or related distress [4]. This moment-to-moment variability can be captured by utilizing ecological momentary assessments [5]. Herein, we consider smartphone-based solutions and apps that can be employed to better understand tinnitus, or to offer assistance to patients with tinnitus in managing tinnitus-related distress.

Interestingly in recent years, smartphones, smartphone apps, and auxiliary health devices such as heart monitors and smart wristbands have gained significant popularity by helping patients to monitor and treat their health problems [6-8]. Specifically, smartphones provide an app ecosystem that can easily be extended with new apps. For instance, on one hand, smartphone-based app solutions can be applied to monitor the ecological or environmental surroundings of patients to better understand health phenomena [9,10]. On the other hand, these solutions can also easily be designed or tailored to assist patients in managing or mitigating the symptoms of their health problems [11]. Within the scope of this paper, such solutions can be directly applied within the context of tinnitus and other closely related health complaints, such as stress [12-15], Ménière disease [16,17], hearing loss [18-20], vertigo [21-23], and dementia [24,25]. Consequently, developers frequently push new apps to the markets to capitalize on the growing interest in health care–related smartphone apps in both academia and industry. Thus, timely assessment and evaluation of these smartphone apps are critically important, particularly due to the sensitive nature of their target domain.

Several mobile health (mHealth) app assessment tools and models are available [26-29], yet they either are focused on a particular health domain or are too time consuming and complex to employ in research. Furthermore, it is often hard to establish an objective score based on these rating and assessment tools. Nonetheless, among them the Mobile Application Rating Scale (MARS) [30] is a reliable and valid instrument for the quality assessment of smartphone-based medical apps or mHealth apps. It offers a straightforward multidimensional rating process to objectively assess health care apps without requiring excessive training for the rater. In addition, MARS has been diversely and widely used in evaluating smartphone-based health apps, for instance, pain management apps [31], weight management apps [32], diabetes management apps [33], and rheumatology apps [34,35]. Additionally, MARS can be employed for rating smartphone apps for complex psychological or physiological conditions such as depression [36], hypertension [37], or tinnitus [38]. In the field of tinnitus, Sereda and colleagues [38] gathered tinnitus management apps based on patient opinion via a web-based survey. Features of the patients’ most cited apps were then analyzed with MARS. We again apply MARS to evaluate tinnitus management apps, yet in this paper, we emphasized systematic search and exploration of the most prominent commercial mobile app platforms to identify the relevant tinnitus apps. Furthermore, we aimed to identify and evaluate recent apps (those newly added since 2019 or not previously identified) in comparison to those previously reviewed [38].

## Methods

### Overview

Our work offers 3 major contributions. First, based on the PRISMA guidelines [39], we systematically searched, screened, and identified smartphone apps aimed at assisting patients with tinnitus. Second, with respect to the objective quality of the smartphone apps and the user experience ratings, all identified apps were critically evaluated and assessed based on MARS. As an added step, we compared our MARS ratings to ratings from other sources and computed interrating agreements. Last, we gathered information for quality ratings of the health apps from various established information platforms, as well as the star ratings from the Google Play Store and the Apple App Store.

### Finding Relevant Apps

In order to generate an exhaustive overview of relevant tinnitus apps, we employed PRISMA guidelines for a systematic search, screening, and identification of the apps.

We performed an open keyword search (Textbox 1) on 2 of the most prominent app markets, namely Google Play and Apple App stores to cover both major mobile platforms (ie, Android and iOS, respectively). Due to the device-specific limitations of apps from different app stores, we did not include app stores such as Amazon App Store, Sony Apps, Samsung Galaxy Apps, Huawei App Store, and LG SmartWorld in our app search workflow. Furthermore, third-party app providers such as Aptoide and F-Droid were not taken into account as they are not considered to be reliable sources because of security issues and their reliance on rooted devices. Rooting is the process of acquiring full system access or administrative control of mobile devices. This process is highly discouraged by device manufacturers and app developers as it may introduce security vulnerabilities [40].

**Figure 1:**
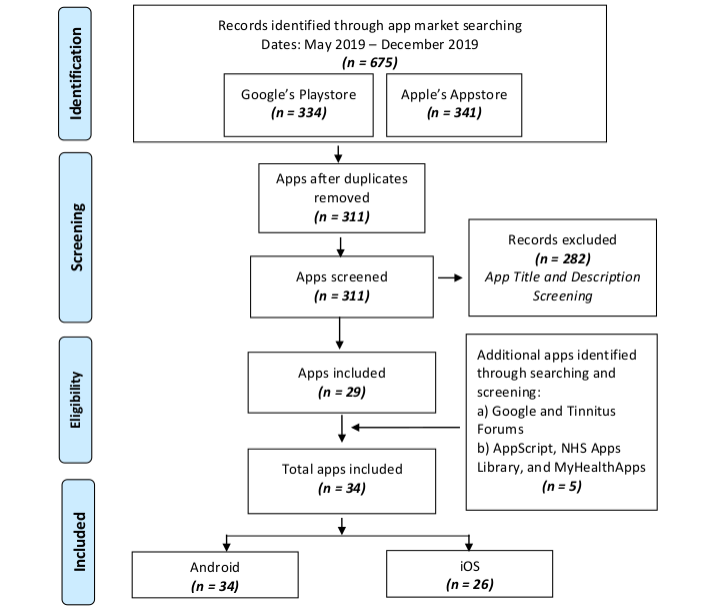
PRISMA workflow for systematic identification of smartphone apps.

The overall workflow to systematically identify relevant apps was based on PRISMA guidelines and is illustrated in Figure 1. The search yielded a total of 675 apps from both app markets (Google Play Store: 334; Apple App Store: 341 apps); 311 apps were identified after removing duplicates. These were screened based on the title and description resulting in 29 apps that satisfied the required criteria.

**Search summary.**

**Sources:** Google Play Store and Apple App Store

**Keywords:***tinnitus*, *hearing*, *noise*, *cognitive behavior therapy (CBT)*, *self-help*

**Strategy:** manual investigation of app title, description, and top 10 comments (if available)

**Inclusion criteria:**

1. Smartphone apps with English title and description

2. Availability of smartphone app on either Google Play Store or Apple App Store

3. App title or description clearly addressing tinnitus, CBT, or self-help as the main subject matter

**Exclusion criteria:**

1. Apps with missing description

2. Misleading apps claiming to address tinnitus, self-help, or CBT.

Using the same keywords (with the keyword *app* appended), Google searches were performed to find any missing or additional apps (in May 2019 and in December 2019). The Google search yielded multiple webpages and forum posts. The contents of these webpages and forum posts were manually investigated to identify additional potentially relevant apps. We also performed searches on 3 independent third-party mHealth app libraries: (1) the government-funded National Health Service Apps Library [41], (2) the privately funded AppScript Library [42], and (3) the privately funded MyHealthApps Cochrane Library [43]. These third-party mHealth app libraries are web portals that curate smartphone apps [44]. This ancillary search of smartphone apps on webpages, tinnitus forums, and third-party mHealth app libraries resulted in the identification of 5 additional apps; therefore, a total of 34 apps were identified for assessment and evaluation. All were available for Android, whereas only 26 apps could also be used on the iOS platform.

### Apps Assessments and Evaluations

The smartphone apps that were identified in the app-store search based on PRISMA guidelines were evaluated using MARS scoring guidelines. To rate the smartphone apps, 4 raters (2 raters from a tinnitus domain, and 2 raters from a mobile app–development domain with background in tinnitus research) were recruited. According to the recommendations of the MARS developers, the raters were instructed to watch a video presentation [45] to familiarize themselves with the scoring process. In addition and to further facilitate the familiarization, the raters were requested to read the MARS scoring paper [30]. Next, all raters rated a sample app for training purposes, and the results were discussed briefly to ensure that all raters had the necessary understanding of the MARS scoring process as well as the individual items of the MARS scale. Finally, all of the raters were assigned all of the identified apps for rating purposes. Note that the quality rating of the MARS is based on a scale ranging from 1 to 5 points: 1=inadequate, 2=poor, 3=acceptable, 4=good, and 5=excellent. MARS further includes 19 items that are divided into 4 subscales, namely, *engagement*, *functionality*, *aesthetics*, and *information quality*. Additionally, MARS includes a fifth category, namely *subjective*, which is not included in the calculation of the final MARS score.

In order to evaluate MARS scores from the 4 raters, we calculated the interrater agreement based on Fleiss κ [46], the internal consistency was based on Cronbach α [47], and the interrater reliability was based on Guttman λ6 [48] as well as intraclass correlation—ICC(2,k) with 95% CI [49].

## Results

### Tinnitus Relief Using Smartphones

A comprehensive list of apps that assist patients (ie, for tinnitus-related relief) and that were identified through PRISMA is shown in Table 1, with their respective properties. Among the app properties, the *downloads* property provides insight into the apps’ usage, however, in the case of the iOS platform, the number of users is not publicly provided by the app store; *rating* provides a general understanding of the quality of the app based on the user opinion and is according to the app store’s rating system; *update* reports the last recorded update for the corresponding store; and for *pricing*, the app price is given, or if the app was free, further information is given.

**Figure 2:**
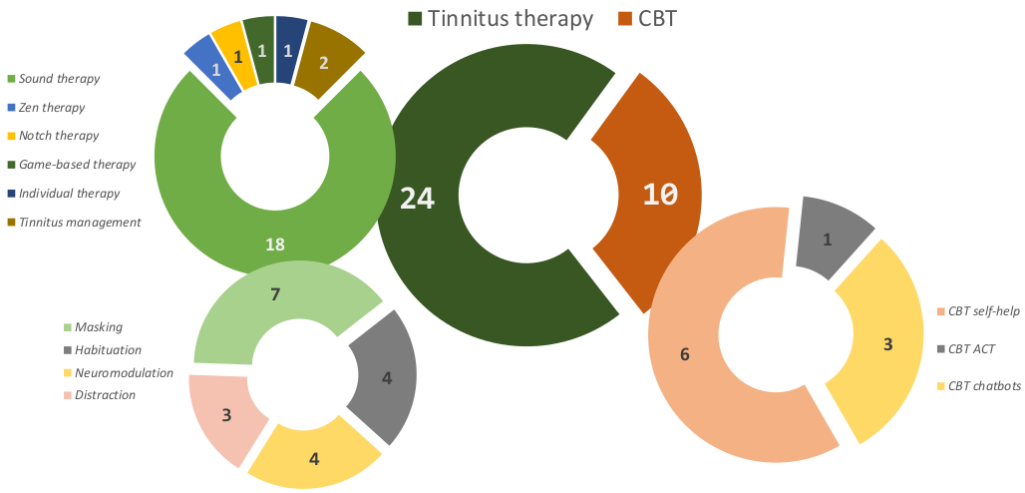
Categorical distribution of identified apps. CBT: cognitive behavior therapy; ACT: acceptance and commitment therapy.

A categorical distribution of the smartphone apps that were identified is depicted in Figure 2. Two main categories were identified based on the app descriptions: tinnitus therapy (24 apps) and cognitive behavior therapy (10 apps). In the tinnitus-therapy category, 18 apps had the main focus of providing of sound therapy, including sounds for tinnitus masking (7 apps), sound habituation (4 apps), neuromodulation (4 apps), and distraction (3 apps). The remaining 6 apps of the tinnitus-therapy category were almost evenly distributed among zen therapy (1 app), notch therapy (1 app), game-based therapy (1 app), individual therapy (1 app), and tinnitus management (2 apps). Cognitive behavior therapy for tinnitus made up the other main category (self-help: 6 app; chatbots: 3 app; acceptance and commitment therapy: 1 app).

**Table 1:** Tinnitus relief–related apps.

App^a^	Description	Platform	Downloads	Rating (out of 5)	Update	Pricing, € (US $)^b^
Beltone Tinnitus Calmer^c^	Combination of relaxation exercise and sound therapy to avoid tinnitus	Android	>1000	4.7	September 2019	In-app purchases
		iOS	—	5.0	September 2019	In-app purchases
CBT^d^ Companion	Employs visual tools to learn and practice CBT techniques	Android	>50,000	4.6	February 2019	In-app purchases
		iOS	—	4.7	February 2019	Free
Diapason for tinnitus^c^	Game-based digital therapeutic providing app for tinnitus relief	Android	>5000	3.1	May 2019	In-app purchases
		iOS	—	—	May 2019	In-app purchases
H & T Sound Therapy	Noise player (pink noise, white noise or brown noise) for masking tinnitus	Android	>10,000	4.3	October 2019	Free
Kalmeda mynoise^c^	Offers medically based individual tinnitus therapy	Android	>1000	3.0	July 2019	In-app purchases
		iOS	—	3.6	July 2019	In-app purchases
MindShift CBT^c^	CBT tools to manage and control anxiety	Android	>100,000	3.9	October 2019	Free
		iOS	—	4.2	October 2019	Free
Moodfit—Stress & Anxiety	Stress and anxiety management and tracking, and offers CBT exercises	Android	>5000	4.4	August 2019	Free
myNoise^c^	Controlling tinnitus via combination of different sounds and noises	Android	>100,000	4.4	March 2018	In-app purchases
		iOS	—	4.6	April 2019	In-app purchases
Quirk CBT	Self-help CBT companion based on 3-column technique	Android	>10,000	3.6	July 2019	In-app purchases
		iOS	—	4.7	September 2019	In-app purchases
Relax Melodies^c^	Sleep assisting app that combines sounds and melodies	Android	>10,000,000	4.6	May 2019	Ad-supported, in-app purchases
		iOS	—	4.8	May 2019	In-app purchases
Relax Noise 3^c^	Masking tinnitus by using red, white, or pink noise	Android	>100,000	4.2	March 2015	Free
ReSound Relief^c^	Avoiding tinnitus using combination of sound therapy and relaxation exercise	Android	>100,000	4.5	February 2019	In-app purchases
		iOS	—	4.7	January 2019	In-app purchases
Sanvello—Stress & Anxiety Help	Audio and video CBT exercises, anxiety tracking and management	Android	>1,000,000	4.6	February 2019	In-app purchases
		iOS	—	4.8	Nov 2019	In-app purchases
SimplyNoise^c^	Controlling and managing stress and tinnitus using white and brown noise	Android	>50,000	3.7	June 2012	Free
		iOS		4.4	May 2018	In-app purchases
Stress & Anxiety Companion	CBT based visual exercises to manage stress and anxiety	Android	>10,000	4.2	July 2019	In-app purchases
		iOS	—	4.6	June 2019	In-app purchases
Starkey Relax^c^	Tinnitus masking, self-management, and education app	Android	>10,000	4.3	October 2017	Free
		iOS	—	3.9	October 2017	Free
StopTinnitus^c^	Masking tinnitus using customized tones	Android	>100	2.7	January 2015	7.95 (9.38)
		iOS	—	1.3	January 2015	8.03 (9.48)
Tinnitracks^c^	Controlling and managing tinnitus by filtering out music for sound therapy	Android	>10,000	3.8	April 2019	In-app purchases
		iOS	—	3.6	February 2019	In-app purchases
Tinnitus Balance App^c^	Controlling annoying tinnitus using customized sounds or music	Android	>50,000	3.7	March 2016	Free
		iOS	—	2.3	March 2016	Free
Tinnitus Help^c^	Tinnitus masking using natural sounds or music	Android	>500	3.0	November 2015	9.90 (11.68)
		iOS	—	4.4	January 2019	17.99 (21.23)
Tinnitus Notch	Provided custom tailored notch therapy for tinnitus relief	Android	>1000	2.7	September 2016	In-app purchases
Tinnitus Peace	Offers melodies to match the frequency of tinnitus to reduce its effects	Android	>5000	3.8	November 2015	Free
Tinnitus Relief	Tinnitus masking using headphones	Android	>10,000	4.2	December 2019	In-app purchases
Tinnitus Sound Therapy	Sound/acoustic therapy for masking tinnitus	Android	>10,000	3.9	June 2019	Free
Tinnitus Therapy (Lite)^c^	Avoiding tinnitus with sound masking and therapy	Android	>500	3.6	February 2019	6.49 (7.66)
		iOS	—	5.0	March 2019	5.36 (6.32)
Tonal Tinnitus Therapy^c^	Helps to mitigate symptoms of tonal tinnitus based on acoustic neuromodulation	Android	>10,000	4.0	July 2018	In-app purchases
Track Your Tinnitus^c^	Managing tinnitus by tracking tinnitus patterns in daily activity	Android	>1000	2.1	October 2018	Free
		iOS	—	5.0	June 2017	Free
What's Up? A Mental Health App	Offers CBT and ACT^e^ methods to manage stress, anxiety as well as depression	Android	>50,000	4.4	June 2019	In-app purchases
		iOS	—	4.6	December 2016	In-app purchases
Whist^c^	Controlling tinnitus using sounds with adjusted volume, pitch, etc	Android	>1000	4.2	March 2017	2.18 (2.57)
		iOS	—	3.7	January 2019	1.78 (2.10)
White Noise (Lite)^c^	Masking and controlling tinnitus using environmental sounds	Android	>5000	4.6	September 2018	3.19 (3.76)
		iOS	—	4.8	April 2019	2.67 (3.15)
Widex Zen^c^	Avoiding tinnitus using relaxing zen sounds and exercises to manage tinnitus	Android	>10,000	3.8	May 2017	Free
		iOS	—	5.0	November 2017	Free
Woebot—Your Self-Care Expert^c^	A chatbot for guided-CBT to manage stress and anxiety	Android	>100,000	4.8	November 2019	Free
		iOS	—	4.7	November 2019	Free
Wysa: Mental Health Therapy^c^	A chatbot offering CBT and DBT^f^ techniques	Android	>1,000,000	4.7	November 2019	In-app purchases
		iOS	—	4.7	December 2019	In-app purchases
Youper—Emotional Health^c^	A chatbot based on CBT and ACT techniques monitoring and tracking mood changes	Android	>1,000,000	4.7	December 2019	In-app purchases
		iOS	—	4.9	December 2019	In-app purchases

^a^Retrieved December 15, 2019.

^b^An exchange rate of €1 to US $1.18 is applicable.

^c^Apps reported in literature.

^d^CBT: cognitive behavior therapy.

^e^ACT: acceptance and commitment therapy.

^f^DBT: dialectical behavior therapy.

### Evaluation of Tinnitus Relief Apps

**Figure 3:**
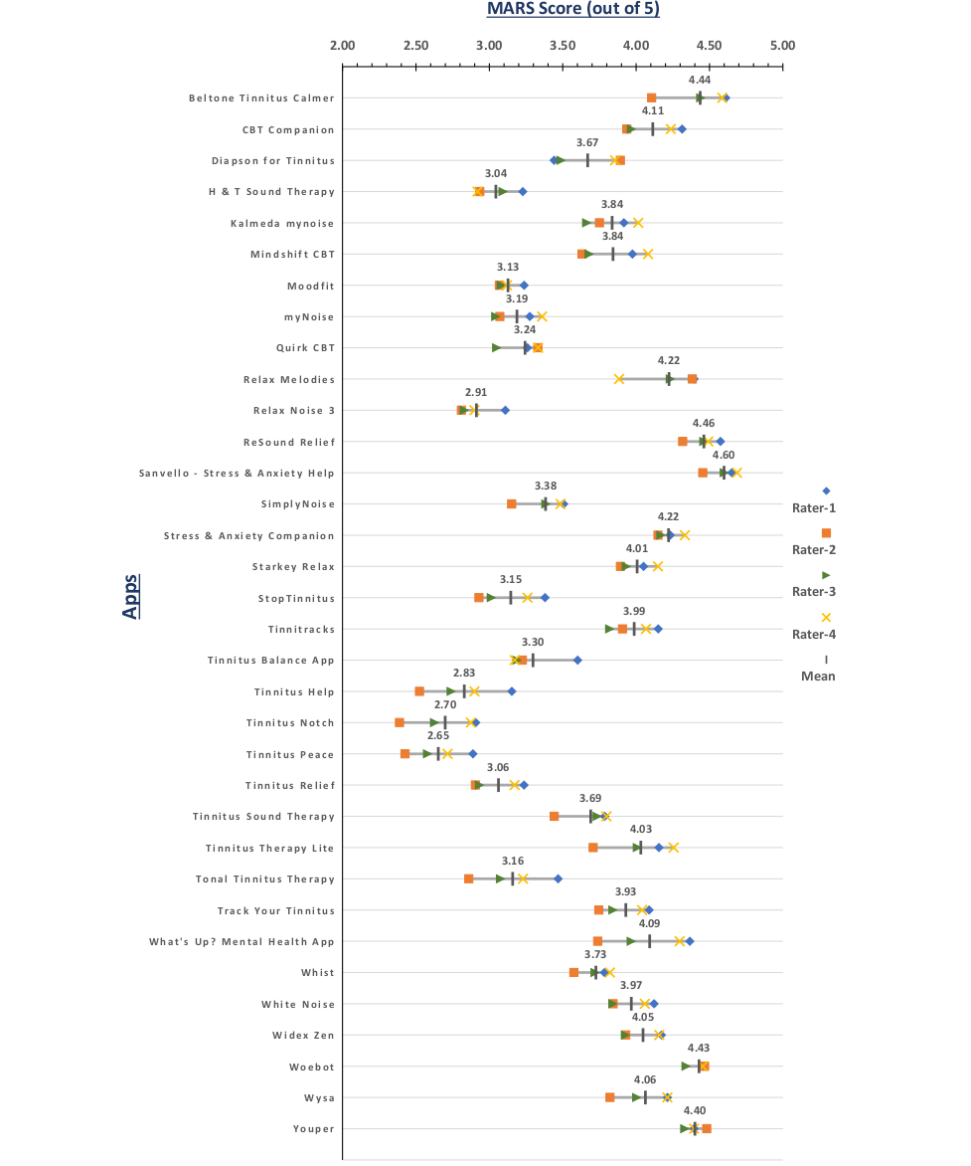
MARS scores.

The ratings of each individual rater and the mean of all 4 raters are depicted in the Figure 3; it can be seen that evaluations are rather consistent between the 4 raters. Note, that MARS ratings range from 1 (inadequate) to 5 (excellent); however, none of the apps in our evaluation process scored less than 2. To ensure consistency between raters and internal consistency, as well as reliability, we performed statistical psychometric analyses (Table 2).

**Figure 4:**
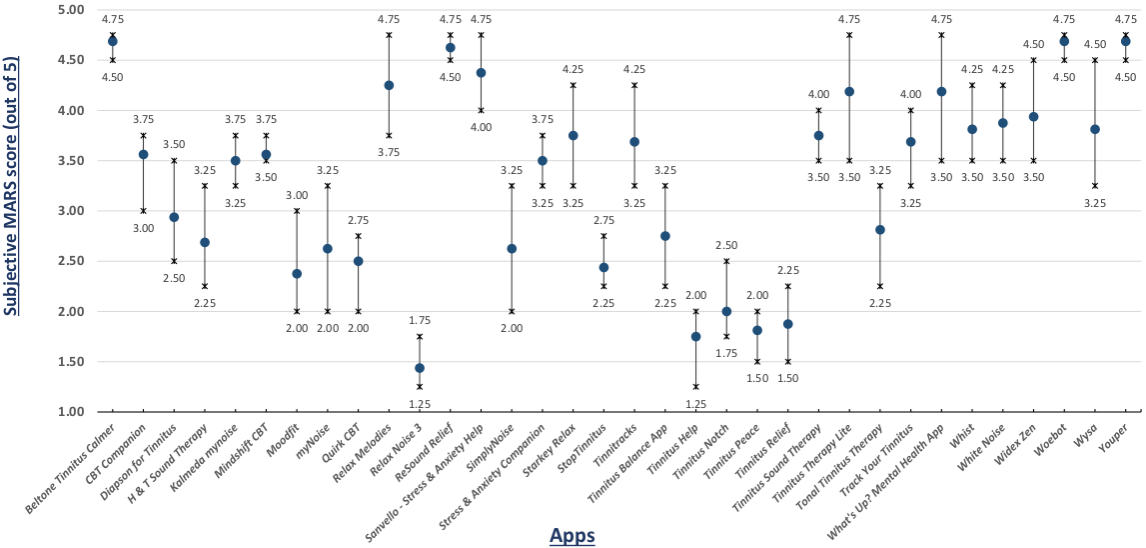
Mean MARS subjective score (with range).

In addition to the objective MARS scores calculated using the arithmetic mean of 4 categories (engagement, functionality, aesthetics, and information quality), MARS guidelines also allow subjective scoring of the smartphone apps, reflecting individual rater opinion. In Figure 4, the results of the subjective criteria of the MARS questionnaire are shown.

**Table 2:** Interrater agreement, internal consistency, and reliability with interpretations [46-49].

Rating category	Fleiss κ	Cronbach α	Guttman λ6	ICC(2,k)^a^ (95% CI)
Engagement	0.62 (substantial)	.93 (excellent)	0.93 (high)	0.92 (0.88-0.95) (excellent)
Functionality	0.52 (moderate)	.90 (excellent)	0.89 (high)	0.89 (0.82-0.93) (good)
Aesthetics	0.49 (moderate)	.96 (excellent)	0.97 (high)	0.94 (0.88-0.97) (good)
Information	0.78 (substantial)	.96 (excellent)	0.96 (high)	0.96 (0.94-0.98) (excellent)
MARS^b^ score	0.74 (substantial)	.95 (excellent)	0.94 (high)	0.94 (0.91-0.97) (excellent)
Subjective score	0.45 (moderate)	.96 (excellent)	0.96 (high)	0.95 (0.92-0.97) (excellent)

^a^ICC: intraclass correlation.

^b^MARS: Mobile Application Rating Scale.

Additionally, we gathered information on the quality of the apps from various repositories. We used existing ratings both from established information platforms for health app quality ratings as well as from the Google Play Store and the iOS App Store (star ratings). The first information platform that we chose was PsyberGuide [50], which is a nonprofit website that is funded by One Mind and operated by Northwestern University. PsyberGuide’s app reviews consist of a *credibility* score that represents the research support, a *user experience* score that is based on MARS, and a *transparency* score that represents the app developer’s privacy information transparency. The second information platform that we chose was ORCHA [51], an organization that offers evaluations of health apps and advice for governments, health, and social care organizations. ORCHA app reviews consist of a score that is a calculated mean of the 3 domains: *data privacy*, *clinical assurance*, and *user experience* plus a level that classifies the app in 1 of 5 levels based on their focus and functionality. This quality information is given in Table 3.

**Figure 5:**
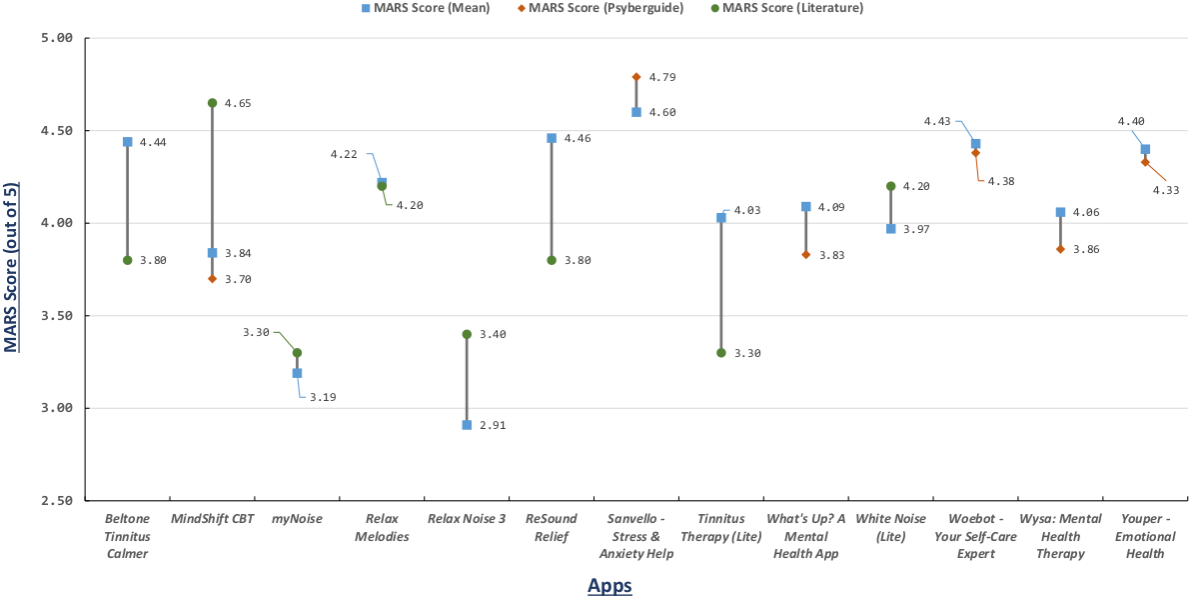
MARS score comparison.

Since the objective MARS scores calculated in this paper and PsyberGuide and ORCHA ratings (apart from PsyberGuide’s user experience) are incomparable, a separate chart (Figure 5) depicts a comparison of MARS scores from our study with those from 2 papers from literature [38,52] and with PsyberGuide’s user experience score. The MARS scores in [38] are for general tinnitus apps such as Beltone Tinnitus Calmer, Relax Noise 3, ReSound Relief, myNoise, Tinnitus Therapy (Lite), and White Noise (Lite), while MARS scores in [52] are given for mindfulness and cognitive behavior therapy apps such as Relax Melodies and MindShift CBT.

**Table 3:** Rating scores comparison (higher numbers are better for all criteria).

App	PsyberGuide			ORCHA			Star rating	
	Credibility	User experience	Transparency	Score, %	Level	Android		iOS
MindShift CBT	2.14	3.70	Questionable	69	3	3.9		4.2
Sanvello—Stress & Anxiety Help	4.29	4.79	Acceptable	N/A^a^	N/A	4.6		4.8
Stress & Anxiety Companion	1.80	N/A	N/A	N/A	N/A	4.2		4.6
What’s Up? A Mental Health App	1.43	3.83	Unacceptable	51	3	4.4		4.6
Woebot—Your Self-Care Expert	4.64	4.38	Acceptable	N/A	N/A	4.8		4.7
Wysa: Mental Health Therapy	3.86	3.86	Acceptable	N/A	N/A	4.7		4.7
Youper—Emotional Health	2.50	4.33	Questionable	N/A	N/A	4.7		4.9
Widex Zen	N/A	N/A	N/A	77	3	3.8		5.0
Whist	N/A	N/A	N/A	71	3	4.2		3.7
Tinnitus Therapy (Lite)	N/A	N/A	N/A	68	3	3.6		5.0
White Noise (Lite)	N/A	N/A	N/A	59	3	4.6		4.8
Tinnitus Relief	N/A	N/A	N/A	56	3	4.2		N/A
Relax Melodies	N/A	N/A	N/A	55	3	4.6		4.8
Beltone Tinnitus Calmer	N/A	N/A	N/A	54	3	4.7		5.0
Resound Relief	N/A	N/A	N/A	54	3	4.5		4.7

^a^N/A: not available.

## Discussion

### Literature

A significant portion of tinnitus scientific literature [53-57] reports on different smartphone and mobile crowdsensing apps to support clinicians in better understanding tinnitus, ranging from data collection to mitigating tinnitus symptoms via therapeutic interventions. These apps are specifically designed to assist patients, clinicians, and researchers alike. From the perspective of patients, these apps aim to provide the necessary means to mask, control, mitigate, or manage tinnitus symptoms. For example, the TrackYourTinnitus smartphone app systematically records data about fluctuations of tinnitus symptoms over time from patients, thus providing information about patient’s tinnitus variability [53]. Similarly, Henry et al [54] delve into the development of an app, based on progressive tinnitus management, to support patients in learning and using coping skills for tinnitus. From the perspective of clinicians and researchers, these apps support a better understanding of tinnitus, particularly in identifying symptom severity and tinnitus characteristics in different patients. For instance, the data collected from the TrackYourTinnitus app can be used to associate tinnitus with daily routines or activities [55] or to shape recruiting strategies for tinnitus-related studies [56]. Similarly, TrackYourTinnitus app was also used to better understand tinnitus variability and tinnitus loudness and stress associations [57].

Generally, health care apps have been exhaustively discussed and reviewed in the literature [58]; however, the number of papers that focus on reviewing and evaluating tinnitus-related smartphone apps is underwhelming. Nevertheless, among pre-existing literature, some papers have discussed the role of tinnitus smartphone apps in clinical scenarios. For instance, internet- and smartphone-based solutions for treatment and management of tinnitus have been reviewed in [59], similarly, the review by Kalle et al [60] discusses internet- or smartphone-delivered cognitive behavior therapy with particular focus on self-help for tinnitus. Both of these papers demonstrate the role of several approaches and technologies involved in advancing tinnitus clinical practice but focus less on current and available apps for patients. Furthermore, the review by Lui et al [61] addresses efficacy or effectiveness of therapeutic solutions provided by mental health apps and hearing health care apps have been discussed in [62]. Comparatively, both [61,62] list a limited number of apps and many are no longer commercially available on the app stores. Specifically, in terms of the assessment of tinnitus-related smartphone apps using MARS, the reviews by Sereda et al [38], which were further extended and repeated by Smith et al [63], are the only closely related works in relation to this work. Both of these reviews [38,63] list tinnitus management apps based on patient opinions, gathered via a web-based survey. The added value of our review was primarily the exploration of smartphone app markets to reveal relevant apps as opposed to using a survey. Additionally, our proposed work also compares the star ratings and MARS scores with quality information gathered by third-party app assessment platforms.

### Limitations

A noticeable limitation of our work was the restricted search of relevant smartphone apps to only 2 app stores. Although the restriction was justified in the paper, it might be possible that there would be benefit in exploring other app stores, such as Amazon and Samsung app stores. Another possible limitation lay in the inclusion criteria for the apps. To include an app, we inspected the app description and a few top-rated comments from the users. Despite being effective and straight-forward, this approach is subjective and highly relies on the knowledge of the inspector about the domain. This limitation can be overcome or can be further improved by gathering additional opinions from domain experts.

### Future Work

In future research, the study will be extended and developed in 2 directions. First, additional app evaluation and assessment instruments (currently under development or newly developed) will be used to repeat the study. For example, recently, the THESIS app evaluation instrument was presented [64]. Therefore, we intend to extend our current work by evaluating the identified apps using THESIS and comparing the results with those of MARS. This will include updates on the available and relevant apps in the app stores. Second, although the already developed instruments systematically and objectively measure the quality of mHealth apps from a user-experience perspective, the lack of instruments to clinically validate smartphone apps is undeniable. Therefore, we intend to further invest our efforts in this research direction. Additionally, as a consequence of this study, we learned that there exist 7 evidence-based tinnitus apps; therefore, we are currently working on a review paper detailing the scientific evidence of these 7 apps.

### Principal Findings

The aim of this study was to systematically identify smartphone apps within the context of tinnitus. The identification process yielded a total of 34 commercially available tinnitus smartphone apps, which were divided in 2 categories: tinnitus therapy (24 apps) and cognitive behavior therapy (10 apps). In an added step, we evaluated the identified apps using MARS. From MARS objective scores (Figure 3), first, we can see that all 34 identified apps have MARS scores higher than 2, indicating that most of these apps provide some level of user experience and that they all have some functional value. Furthermore, the MARS rating process discovered that only 7 apps—Tinnitus Therapy (Lite), ReSound Relief, SimplyNoise, Audio Notch, Wysa, Woebot, and MindShift—out of 34 apps scored in the evidence-based subitem of the information category, suggesting a lack of clinical validation for most of the apps. Furthermore, the mean MARS scores (Figure 3) of all 4 raters ranged from 2.65-4.60, with Tinnitus Peace having the lowest mean score (mean 2.65, SD 0.20) and Sanvello—Stress and Anxiety Help having the highest mean score (mean 4.60, SD 0.10). On the individual-rater level, Tinnitus Notch had the lowest (score: 2.39), while, Sanvello—Stress and Anxiety Help had the highest (score: 4.69). Furthermore, some apps were rated better in comparison to the others. For instance, Beltone Tinnitus Calmer, Resound Relief, Sanvello—Stress and Anxiety Help, as well as Woebot and Youper received very good ratings, whereas Tinnitus Help, Tinnitus Notch, and Tinnitus Peace were the worst of all. The mean subjective scores presented in Figure 4 ranged from 1.44-4.69, with Relax Noise 3 having the lowest scores (mean 1.44) and Beltone Tinnitus Calmer, Woebot, and Youper having the highest scores (mean 4.69). From Figure 4, naturally, the range of the subjective scores was wider than those of the objective scores; however, it is notable that the mean subjective results were more or less in line with the mean objective results.

Values from the 4 psychometric measures (Table 2) confirm the reliability and validity of our MARS rating procedures, especially for measures testing internal consistency and reliability (>0.89). The interrater agreement, as demonstrated by Fleiss κ, was merely moderate for subjective, functionality, and aesthetics scores. This is noteworthy and may be related to individual differences in the raters with regards to their backgrounds. In any case, from Figure 3, Figure 4, and Figure 5, it is evident that our rating procedures generally produced valid, reliable, and thus viable results.

Interestingly, the expert ratings from both information platforms (PsyberGuide and ORCHA) and the user ratings from the app stores varied, sometimes considerably. For example, What’s Up? A Mental Health App received high ratings from its users in the app stores (Google Play Store: 4.4; Apple App Store: 4.6), whereas both expert ratings were considerably lower (PsyberGuide: credibility: 1.43, transparency: unacceptable, user experience: 3.38; ORCHA: 51%). Another app whose app store rating differed significantly from its ORCHA rating is Beltone Tinnitus Calmer (Google Play Store: 4.7; Apple App Store: 5.0; ORCHA: 54%). The ORCHA score was moderate as a result of a moderate rating for data privacy and clinical assurance; the apps hadn’t been rated by PsyberGuide. These examples illustrate that an independent rating with validated instruments is crucial for the informed selection of health apps.

From Figure 5, it can be seen that the MARS scores in our work were more in line with those of PsyberGuide’s user experience score. Similarly, the differences between MARS scores from our paper and those from literature were evidently higher for all apps except myNoice, Relax Melodies, and White Noise (Lite). These differences were as a result of version changes in the apps. In our case, similar to PsyberGuide’s case, the MARS scores present the contemporary assessment of the most recent version of the apps, thus validating the need of an up-to-date MARS assessment of tinnitus apps.

All 34 identified apps obtained a MARS objective score higher than 2 (ranging between 2.65-4.60), indicating that they provide some level of user experience and at least some technical functional value for the user. Furthermore, in addition to presenting the objective MARS scores, the subjective MARS scores (ranging between the values of 1.44-4.69) were also discussed. The 4 psychometric measurements—Fleiss κ, Cronbach α, Guttman λ6, and ICC(2,k)—confirmed and depicted positive interrater agreement, internal consistency as well as reliability between the raters. The only exception was noticed in Fleiss κ with moderate values for subjective score, functionality, and aesthetics. The quality information of identified apps from PsyberGuide and ORCHA as well as the star ratings from the Google Play Store and the Apple App Store were compared. The quality information comparison exhibited incongruity. Finally, the comparison between MARS scores from this work and MARS scores of smartphone apps reported in previously published papers depicted a high coherence. Through these steps, we were able to comprehensively capture the wide array of heterogeneous apps for tinnitus and present an up-to-date assessment of identified apps.

### Conclusions

This work highlighted the impact of smartphone apps, specifically within the context of tinnitus research. As a consequence, we demonstrated that there exists a plethora of smartphone apps utilized in supporting and controlling tinnitus symptoms, understanding tinnitus, and monitoring patients with tinnitus. Among the 34 identified apps, only 7 apps were evidence-based suggesting that the majority were in need of more stringent clinical validation.

## Abbreviations

ICC: intraclass correlation
MARS: Mobile Application Rating Scale
mHealth: mobile health
